# Kidney Cancer Models for Pre-Clinical Drug Discovery: Challenges and Opportunities

**DOI:** 10.3389/fonc.2022.889686

**Published:** 2022-05-10

**Authors:** Laura Pohl, Jana Friedhoff, Christina Jurcic, Miriam Teroerde, Isabella Schindler, Konstantina Strepi, Felix Schneider, Adam Kaczorowski, Markus Hohenfellner, Anette Duensing, Stefan Duensing

**Affiliations:** ^1^ Molecular Urooncology, Department of Urology, University Hospital Heidelberg, Heidelberg, Germany; ^2^ Department of Urology, University Hospital Heidelberg and National Center for Tumor Diseases (NCT) Heidelberg, Heidelberg, Germany; ^3^ Precision Oncology of Urological Malignancies, Department of Urology University Hospital Heidelberg, Heidelberg, Germany; ^4^ Cancer Therapeutics Program, UPMC Hillman Cancer Center, Pittsburgh, PA, United States; ^5^ Department of Pathology, University of Pittsburgh School of Medicine, Pittsburgh, PA, United States

**Keywords:** renal cell carcinoma, intratumoral heterogeneity (ITH), drug development, patient-derived xenografts (PDX), preclinical studies

## Abstract

Renal cell carcinoma (RCC) is among the most lethal urological malignancies once metastatic. The introduction of immune checkpoint inhibitors has revolutionized the therapeutic landscape of metastatic RCC, nevertheless, a significant proportion of patients will experience disease progression. Novel treatment options are therefore still needed and *in vitro* and *in vivo* model systems are crucial to ultimately improve disease control. At the same time, RCC is characterized by a number of molecular and functional peculiarities that have the potential to limit the utility of pre-clinical model systems. This includes not only the well-known genomic intratumoral heterogeneity (ITH) of RCC but also a remarkable functional ITH that can be shaped by influences of the tumor microenvironment. Importantly, RCC is among the tumor entities, in which a high number of intratumoral cytotoxic T cells is associated with a poor prognosis. In fact, many of these T cells are exhausted, which represents a major challenge for modeling tumor-immune cell interactions. Lastly, pre-clinical drug development commonly relies on using phenotypic screening of 2D or 3D RCC cell culture models, however, the problem of “reverse engineering” can prevent the identification of the precise mode of action of drug candidates thus impeding their translation to the clinic. In conclusion, a holistic approach to model the complex “ecosystem RCC” will likely require not only a combination of model systems but also an integration of concepts and methods using artificial intelligence to further improve pre-clinical drug discovery.

## Introduction

Metastatic renal cell carcinoma (RCC) is among the most lethal urological malignancies (1, 2). These tumors are largely resistant to conventional chemo- or radiotherapy but vulnerable to immune modulatory treatment and agents that target the VEGF/VEGFR axis (3). The notion that spontaneous remissions can occur in RCC has spurred efforts to harness the patient’s antitumoral immune response (4, 5). Initially, this was attempted by using immune stimulatory cytokines such as interleukin-2 alone or in combination with interferon-alpha (6). This early form of immunotherapy was found to lead to remarkably durable responses, however, only in a relatively small subgroup of patients (7). Nevertheless, this observation has stimulated more in-depth research into the mechanisms of immune evasion that culminated in the development of immune checkpoint inhibitors (ICIs) (8). The period between the cytokine and the ICI eras was dominated by VEGF/VEGFR targeting agents, most prominently tyrosine kinase inhibitors (TKIs) such as sunitinib (9). This treatment approach was based on a better understanding of the basic biology in particular of clear cell RCC (ccRCC), which commonly harbors an inactivation of the *VHL* gene and deregulation of the HIF/VEGF transcriptional network (3). Currently, combination therapies of ICIs and TKIs such as pembrolizumab and axitinib are the standard of care for patients with metastatic ccRCC (10). Despite the recent innovations in the therapeutic landscape, a significant proportion of patients will experience disease progression within a year or two after the initiation of systemic treatment (11). Hence, there is still an urgent need for new approaches to treat patients with advanced RCC. As highlighted in this Special Issue, suitable model systems are pivotal for successful pre-clinical drug development. At the same time, RCC is characterized by several peculiarities that need to be taken into consideration when using and developing RCC model systems.

## RCC Subtypes: One Size Does Not Fit All

While ccRCC represents the most common form of kidney cancer (approximately 75%), several other subtypes with non-clear cell RCC (nccRCC) histology such as papillary (approximately 10-20%) or chromophobe (approximately 5-7%) RCC exist. In fact, the 2016 WHO classification of tumors of the kidney lists more than 50 entities including 15 entities of renal cell tumors besides ccRCC (12). Among the newly included renal cell tumors in the 2016 WHO classification were some entities with specific molecular alterations such as fumarate hydratase (FH)-deficient RCC, succinate dehydrogenase (SDH)-deficient renal carcinoma and others such as tubulocystic RCC, clear cell papillary RCC and acquired cyctic disease-associated RCC (12, 13). There is a remarkable and still emerging diversification in the classification of papillary RCC that goes beyond the “classical” type 1 and type 2 subtypes (13). Emerging variants of papillary renal tumors are for example, papillary renal neoplasm with reversed polarity, biphasic squamoid/alveolar RCC, biphasic hyalinizing psammomatous RCC or thyroid-like follicular RCC (14). There are also RCCs that are defined by gene translocations such as the MiT family translocation RCCs (harboring *TFE3* or *TFEB* translocations) or *ALK*-translocated RCCs, which frequently show aggressive growth characteristics (14).

Since nccRCCs are relatively rare, only a limited number of randomized, prospective clinical trials exist (15). Treatment modalities that have been extrapolated from ccRCC such as VEGF- or mTOR-targeting agents have been explored in metastatic nccRCC, however, with suboptimal results (16, 17). A randomized phase II trial has shown a progression-free survival benefit with the MET inhibitor cabozantinib over sunitinib in patients with metastatic papillary RCC, in which *MET* alterations are enriched (18). In line with this finding, the MET inhibitor savolitinib was found to show efficacy in MET-driven papillary RCC (19). Recent phase II trials that explore immune checkpoint inhibitors alone or in combination with cabozantinib in nccRCC show promising activity in patients with papillary RCC but at the same time highlight the therapeutic problem of chromophobe RCC (20, 21). Most nccRCC cell line and patient-derived xenograft (PDX) models have been established for the “classical” papillary RCC (22) and there is an urgent need to further expand model systems to other, less common nccRCC entities, including chromophobe RCC, for a further optimization and individualization of pre-clinical drug discovery.

## Genomic and Functional Intratumoral Heterogeneity (ITH)

In a seminal study published in 2012, metastatic ccRCC was shown to be characterized by a high degree of genomic ITH with indications for evolutionary processes with clonal (“truncal”) and subclonal mutations (23). These findings were later conceptualized in a new classification of ccRCC based on evolutionary subtypes (24). The proposed seven subtypes of ccRCC show distinct combinations of clonal and subclonal alterations including a sequence of mutational events (24). PDX models of RCC have been suggested to reflect, at least to a certain degree, the ITH found in the tumor of origin (25, 26). While it is conceivable that truncal driver aberrations in genes such as *VHL* or *PBRM1* are preserved, it remains unclear whether PDX models can reflect the entire spectrum of subclonal driver mutations due to an inevitable sampling bias. Subclonal drivers can not only promote tumor expansion but have also been implicated as a therapeutic target or in the development of drug resistance (27). Moreover, subclonal as well as certain clonal drivers have been reported to show a regional variability (28). Hence, models of RCC that rely on regional sampling of the patient tumor are likely to underestimate the number and spectrum of driver events. This shortcoming may lead to an overestimation of drug efficacy and an underestimation of drug resistance. In this context, it is noteworthy that some widely used ccRCC cell line models do not harbor inactivated *VHL* (29) and may hence rather rely on subclonal driver events.

One idea to overcome, at least in part, the problem of ITH is the use of liquid biopsies (30). However, it is currently not entirely clear whether and to what extent circulating tumor cells or tumor-derived circulating DNA represent the whole subclonal architecture of RCC. Despite some promising results, principal issues such as low levels of circulating tumor DNA may preclude a wider use of liquid biopsies to characterize ITH in RCC (31).

A number of studies have analyzed the genomic composition or matched primary and metastatic RCC samples (23, 32, 33). There are very few, if any, patient-derived models of matched primary and metastatic sites. However, given the observed genetic discordance between primary tumor and metastases (33), such models would be highly desirable, in particular in patients with synchronous metastatic dissemination.

In addition to genomic ITH, functional ITH also needs to be taken into consideration when modeling RCC (34). One manifestation of functional ITH in ccRCC is the enhanced proliferation and intracellular signaling activity at the tumor invasion front (35). While no specific mutations could be pinpointed to explain this finding, some observations suggest that factors from the tumor microenvironment may be involved (36). The tumor periphery may hence represent a spatial niche that harbors tumor cells with an increased fitness or potentially higher aggressiveness. A recent study used computational modeling to suggest that such a “surface growth” pattern could be associated with an enhanced subclonal diversification (37). While this computational study did not include the microenvironment as a factor that may influence tumor growth, it nevertheless underscores that tumor evolution and growth patterns are very likely connected. It will be important to show whether and to what extent pre-clinical RCC models can reflect this interrelation.

Recently, synthetic DNA barcode tracking and single cell transcriptomics have revealed the existence of spatially confined clonal expansion in PDX models (38). Clonal dominance was driven by differential gene expression, which may not only influence the adaptation to the host microenvironment but also PDX engraftment efficiency in general. Consequently, tumor cell engraftment is subject to selection processes that can ultimately affect the cellular composition of PDXs and therefore drug vulnerabilities.

## Impact of the Tumor (Immune) Microenvironment

Besides a remarkable inter- and intratumoral genomic and functional heterogeneity (23, 35), ccRCC shows a number of immunological features that distinguish it from other tumor entities and that may impose challenges for modeling RCC *in vitro* and *in vivo*. First, a high number of tumor-infiltrating lymphocytes, in particular T cells, is associated with a poorer prognosis in RCC and not with a more favorable outcome as in most other malignancies (39–42). Conversely, immune excluded tumors were generally found to be associated with a better patient prognosis (43). Tumor-infiltrating T cells have been found to be frequently exhausted and to express high levels of inhibitory checkpoint proteins (44). The exhaustion of cytotoxic T cells was found to increase with disease progression together with a reduced clonotypic diversity indicating antigen exposure (45). T cell exhaustion in more advanced ccRCCs appears to involve interactions with immunosuppressive M2 macrophages and not, like in other cancers, regulatory T cells (45). In fact, a vicious cycle has been proposed in which terminally exhausted CD8-positive T cells drive M2 polarization, which, in turn, further promotes T cell exhaustion (45). These findings underscore that malignant tumors including ccRCCs initially represent complex and dynamic ecosystems, in which certain cellular interactions are fostered that later lead to an “ecological collapse” with a breakdown of immune cell diversity and function thus promoting tumor progression.

Besides T cells, recent findings highlight the important role of B cells in antitumor immunity (46). B cells produce antibodies and present antigens to T cells, which involves the formation of tertiary lymphoid structures (TLSs). Tumors with enhanced TLSs have in general a more favorable prognosis (47), which may also be the case in RCC (48). The development of non-clinical RCC models that reflect immune cell exhaustion, T cell-macrophage interactions and TLS formation will remain a daunting task.

While the tumor immune microenvironment conceivably plays an important role in shaping tumor progression, non-immune cells may likewise support clonal and subclonal expansion. Growth factors and cytokines secreted by non-immune cells of the tumor microenvironment with stimulatory effects on tumor growth include FGF-2, among others (36, 49, 50). Efforts to co-culture RCC cells with non-malignant cells of the microenvironment are therefore laudable, however, the functional state of such cells may not adequately reflect the situation *in vivo*, where cells of the tumor microenvironment may become re-programmed by numerous cellular interactions as well as extrinsic insults such as inflammation and necrosis (51). Together, these findings highlight the complexity of microenvironmental cell-cell interactions in RCC that remain a challenge to RCC model development.

## Pre-Clinical Drug Development and the Problem of “Reverse Engineering”

*In vitro* drug screening has long been an important approach to characterize therapeutic vulnerabilities of cancer cells. Conceivably, the success of a drug screen depends on numerous factors including the source of tumor cells, the presence or absence of cells representing the tumor (immune) microenvironment, culture and growth conditions, drug availability, and read-out techniques (52). The high drop-out rate associated with cell-based pre-clinical drug discovery has been attributed to confounding factors of the experimental set-up that leads to an unsatisfying replicability and reproducibility (53). Organoids have been established for a number of human malignancies, however, reports on using organoids for drug screening purposes in RCC are relatively scarce (54–56). In particular patient-derived air-liquid interface organoid models appear to be promising for pre-clinical drug discovery since they maintain features of the original tumor including components of the microenvironment (57, 58). Ultimately, a systemic collection of model systems may help to overcome some problems in pre-clinical drug discovery (58, 59).

Nevertheless, one issue that appears to be quite common is the problem of “reverse engineering” i.e., the inability to characterize the mode of action of a drug that was identified as a hit in a screen. This could be related to unknown drug activities or reflect complex tumor cell-intrinsic molecular interactions and adaptation processes. For example, our group identified the MET inhibitor crizotinib in a compound screen using a primary patient-derived ccRCC cell line with a *MET* missense mutation (R988C) (60). This mutation has been suggested to function as a driver mutation in lung cancer (61, 62). Perplexingly, further experiments showed that *MET* was not a driver in the patient-derived cell line and the mode of action of crizotinib very likely involved its function to block the transmembrane transporter P-glycoprotein (60). A broader implication of this finding is that candidate driver events may not necessarily represent the prime target for tumor cell eradication and that there could be a disconnection between a genotype and the observed phenotype. It is furthermore possible that driver mutations vary with respect to their potency i.e., there could be strong drivers and weak drivers depending on the cellular context and positive or negative driver cooperativity.

## Artificial Intelligence (AI) and Pre-Clinical RCC Models

**Figure 1:**
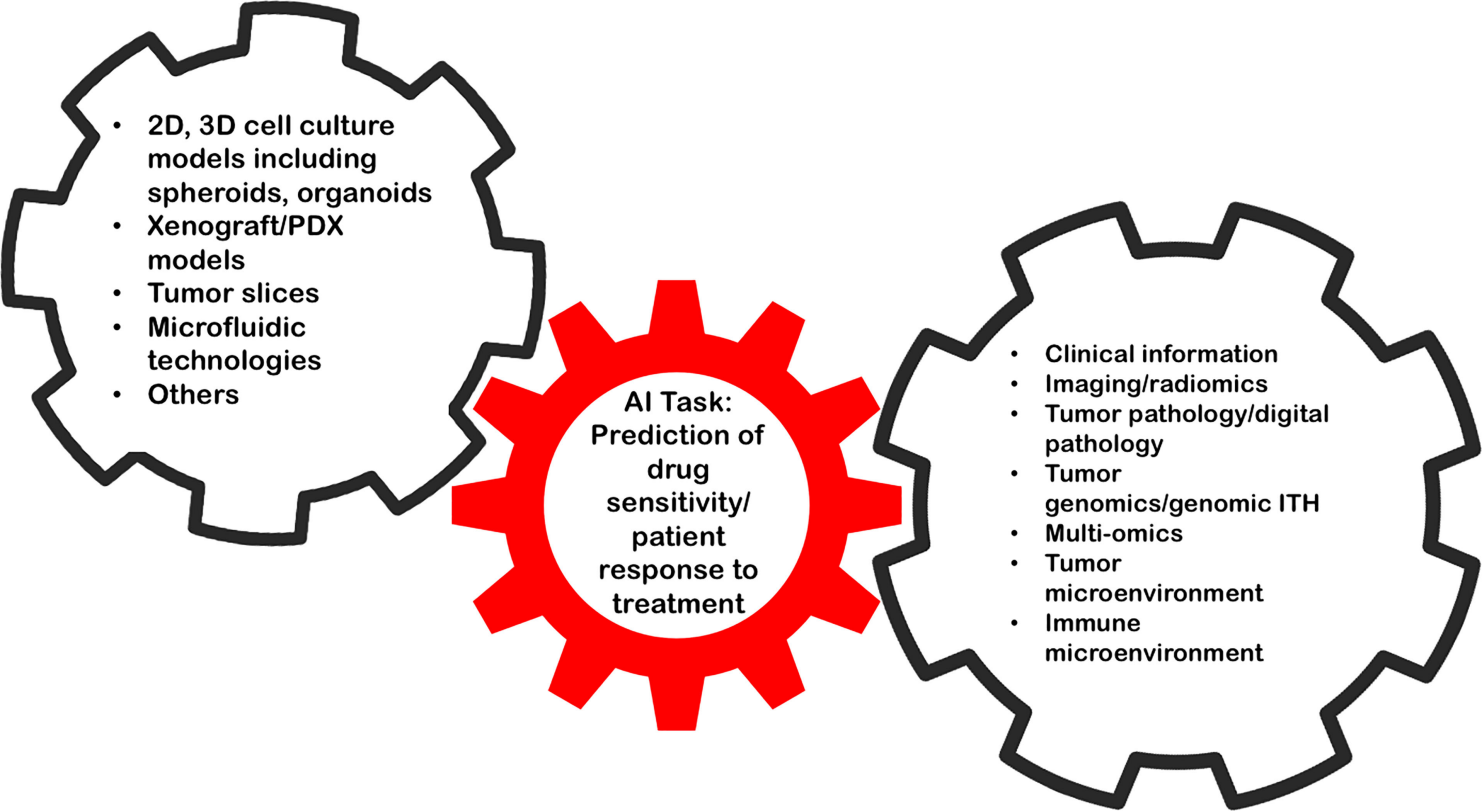
AI holds the promise to link data from RCC model systems to complex and comprehensive clinical data to improve pre-clinical drug discovery.

The high degree of inter- and intratumoral heterogeneity represents a major obstacle for predicting therapeutic responses in patients with advanced RCC (63, 64). This problem also applies to multimodal treatment strategies e.g., the inclusion of surgical interventions during a patient’s path through the disease. Given the intrinsic difficulty to faithfully mirror a tumor ecosystem in its entirety, AI may help to connect pre-clinical RCC models with clinical data and *vice versa* as envisioned in **Figure 1**. Tools to estimate genetic ITH have been developed (65) and may be integrated into such AI algorithms.

There are currently two major AI concepts, one that has a comprehensive view on patient data, e.g. the “digital twin” and a second consisting of highly focused decision-support tasks that frequently use deep learning applications and pattern recognition algorithms (66). AI has the potential to support virtually all aspects of pre-clinical drug discovery (52, 67). For example, machine learning approaches have been developed to aid target/driver gene identification (68). Structure- or ligand-based virtual screening can significantly improve speed and efficacy of lead compound development. Lastly, AI tools can also help to predict pharmacokinetic properties of a new agent (67). It is important to mention that drug-related adverse events in a mostly palliative situation need special consideration. AI and pre-clinical models, for example for cardiovascular toxicity, may help not only to predict adverse events but also to develop protective strategies (67, 69).

These findings underscore the role of AI in the pre-clinical drug development process. Nevertheless, in precision oncology, the global question of “drug sensitivity” of a patient with advanced RCC may be too broad and may need to be subdivided into “narrow-task” questions (66) such as response to a given experimental drug, immune oncological agents, TKIs or others. Ultimately, prospective clinical trials will be needed to determine the impact of AI on patient outcome.

## Conclusion and Outlook

Pre-clinical models are vital to further develop the therapeutic landscape for metastatic ccRCC, yet there are a number of biological peculiarities that may represent serious obstacles for the identification of novel immunological and non-immunological therapies. One approach to overcome these hurdles could be to combine different modeling approaches such as 2D and 3D cell culture models and PDXs (70) despite the difficulty to adequately incorporate immune responses. Moreover, AI has the potential to transform pre-clinical drug development by combining data from non-clinical models with comprehensive patient data including multi-omics analyses. Still, the magnitude and complexity of cellular and molecular interactions that characterizes the “ecosystem RCC” will continue to challenge future generations of tumor biologists.

## Author Contributions

Conceptualization of the manuscript, contributing ideas and information: all authors. Writing the first draft of the manuscript: SD. Critical reading of the manuscript: all authors. All authors contributed to the article and approved the submitted version.

## Funding

Bundesministerium für Wirtschaft und Klimaschutz.

## Conflict of Interest

The authors declare that the research was conducted in the absence of any commercial or financial relationships that could be construed as a potential conflict of interest.

## Publisher’s Note

All claims expressed in this article are solely those of the authors and do not necessarily represent those of their affiliated organizations, or those of the publisher, the editors and the reviewers. Any product that may be evaluated in this article, or claim that may be made by its manufacturer, is not guaranteed or endorsed by the publisher.
